# The Effect of Specimen Size on the Results of Concrete Adiabatic Temperature Rise Test with Commercially Available Equipment

**DOI:** 10.3390/ma7127861

**Published:** 2014-12-08

**Authors:** Byung Jae Lee, Jin Wook Bang, Kyung Joon Shin, Yun Yong Kim

**Affiliations:** 1 Research & Development Center, JNTINC Co. Ltd., Hwaseong 445-842, Korea; E-Mail: lbjae80@gmail.com; 2 Department of Civil Engineering, Chungnam National University, Daejeon 305-764, Korea; E-Mails: best6516@naver.com (J.W.B.); kjshin@cnu.ac.kr (K.J.S.)

**Keywords:** heat of hydration, adiabatic temperature rise test, specimen size, low heat Portland cement, ternary blended cement, early strength low heat blended cement

## Abstract

In this study, adiabatic temperature rise tests depending on binder type and adiabatic specimen volume were performed, and the maximum adiabatic temperature rises and the reaction factors for each mix proportion were analyzed and suggested. The results indicated that the early strength low heat blended cement mixture had the lowest maximum adiabatic temperature rise (*Q*_∞_) and the ternary blended cement mixture had the lowest reaction factor (*r*). Also, *Q* and *r* varied depending on the adiabatic specimen volume even when the tests were conducted with a calorimeter, which satisfies the recommendations for adiabatic conditions. Test results show a correlation: the measurements from the 50 L specimens were consistently higher than those from the 6 L specimens. However, the *Q*_∞_ and *r* values of the 30 L specimen were similar to those of the 50 L specimen. Based on the above correlation, the adiabatic temperature rise of the 50 L specimen could be predicted using the results of the 6 L and 30 L specimens. Therefore, it is thought that this correlation can be used for on-site concrete quality control and basic research.

## 1. Introduction

Mass concrete structures experience restrained stress due to heat of hydration, which is generated during concrete placement. This restrained stress can induce thermal cracks that are harmful to structures [1,2,3,4,5,6]. Thus, for mass concrete structures, the risk of thermal cracking should be considered carefully to prevent unexpected cracks forming. Finite elements analysis can be one of the methods for this purpose. By performing an finite elements analysis of hydration heat and thermal stress, the possibility of thermal cracks during construction processes can be evaluated in the designing and placement planning stages. The factors that affect these cracks include the adiabatic temperature rise of concrete, thermal conductivity, specific heat, convection coefficient, placement temperature, and ambient temperature [7]. In particular, the adiabatic temperature rise of concrete is the most significant factor in regard to the result of the analysis of thermal cracks [1,2,3,4].

The basic method that can be used to avoid or reduce these harmful effects is to adapt a cement with a hydration heat that is relatively lower than that of ordinary cement. Type IV low heat Portland cement (LPC) and blended cement had been used frequently for the construction of mass concrete structures [2,8,9]. In recent years, various binders have been studied and used to control thermal cracks caused by the heat of hydration. The representative binders that are commonly used include ternary blended cement (TBC) and early strength low heat blended cement (EBC) [10]. TBC is a binder that blends type I ordinary Portland cement (OPC) with blast furnace slag (BFS) and fly ash (FA), which are industrial byproducts [11]. EBC is a binder that blends high Blaine BFS, anhydrite, and an alkaline activator to supplement the early strength development of TBC.

Basic and general information about these mixtures can be found in the literature or specifications. The existing Korean Concrete Standard Specification (Chapter 18, mass concrete) suggests the standard values for estimating the adiabatic temperature rises of ordinary, moderate heat, high early strength, blast furnace slag, and fly ash cement mixtures. However, this information can give only a rough estimation, not a precise prediction for the mixture that will be used. The precise thermal characteristics of each mixture cannot be known without a test. Therefore, adiabatic temperature rise tests need to be performed to investigate the characteristics of hydration heat and further evaluate the potential of thermal cracks.

Since the measurement of heat is a convenient way to monitor and quantify the magnitude and the rate of reaction, several types of equipment are used in the material industry and science fields. Two types of heat tests are frequently used in cement science and technology, isothermal hydration and adiabatic hydration [12]. In the isothermal hydration test, the rate of heat evolution is measured from the sample, kept at a constant temperature in the presence of hydration heat. As the size of specimens is limited, this test is usually applied to small paste samples. There are many calorimeters available commercially. The other is an adiabatic test method. This method uses calorimeters for measuring the temperature rise in a sample being hydrated without heat exchanges with the external medium [12]. Adiabatic calorimeters have been developed to measure the temperature rise in cement paste, mortar, and concrete samples.

On the other hand, there are no standards or guidelines related with details of adiabatic temperature rise tests in the world. Only minimum requirements are given in a few specifications or recommendations. Rilem TC 119-TCE defines adiabatic condition as being when the temperature loss of a sample not greater than 0.02 K/h and recommends that the volume of sample be about 4 L to incorporate maximum aggregate size [12]. Therefore, many researchers have designed their own systems, which differ from each other as follows: G. D. Schutter’s: φ280 × h400 mm (24.6 L), M. Liwu’s: φ150 × h300 mm (5.3 L), and I. Tanaka’s: 55 L [13,14,15,16,17].

In Korea, the concrete standard specification, which was revised in 2009, only specifies that adiabatic specimen volume needs to be more than 50 L [2]. However, as the volume of the mix concrete is large, it is difficult to apply research stages or on-site quality control. From the view of convenience and economy, the small volume of the adiabatic apparatus is preferred. Therefore, as an alternative, a test apparatus that adapts an adiabatic specimen volume of 6 L has been increasingly used. However, it is known that the measured adiabatic temperature rise is relatively smaller than that of a 50 L apparatus [18]. Thus, an examination of adiabatic temperature rises depending on adiabatic specimen volume is needed.

Therefore, the purpose of this study is to examine the effects of binders and specimen size on the amount of adiabatic temperature rises so that effective examination of hydration heat is feasible in a small-scale test.

## 2. Experimental Plan and Method

### 2.1. Experimental Plan

Table 1 summarizes the experimental plan for examining the adiabatic temperature rise of concrete depending on binder type and adiabatic specimen volumes. For the binder, OPC, LPC, TBC, and EBC were used, respectively. The unit weight of binder was set to three levels (300, 400, and 500 kg/m^3^). Also, for a constant placement temperature of concrete, the materials were stored and cured in a constant temperature room (20 °C). The target slump and the target air content were set to 150 ± 25 mm and 4.5% ± 1.5%, respectively. For the chemical admixture, the amount was adjusted so that the target slump and air content could be satisfied.

### 2.2. Materials and Mixing

Table 2, Table 3, Table 4 summarizes the physical and chemical properties of the materials. For the LPC mixture, the product from Ssangyong Cement Company (Yeong wol-gun, Korea) was used; and for the OPC, TBC, and EBC mixtures, the products from Hanil Cement Company (Dan yang-gun, Korea) were used. For the fine aggregate, river sand with a saturated surface dry density of 2.59 and a water absorption ratio of 1.57% was used; and for the coarse aggregate, crushed stone (25 mm) with a fineness modulus of 7.06 and a water absorption ratio of 0.40% was used. For the OPC mix proportioning, a polynaphthalene sulfonate-based standard AE water reducing agent with a specific gravity of 1.20 was used; and for the LPC, TBC, and EBC mix proportioning, a polycarboxylic acid-based high-range water reducing agent with a specific gravity of 1.05 was used.

**Figure 1:**
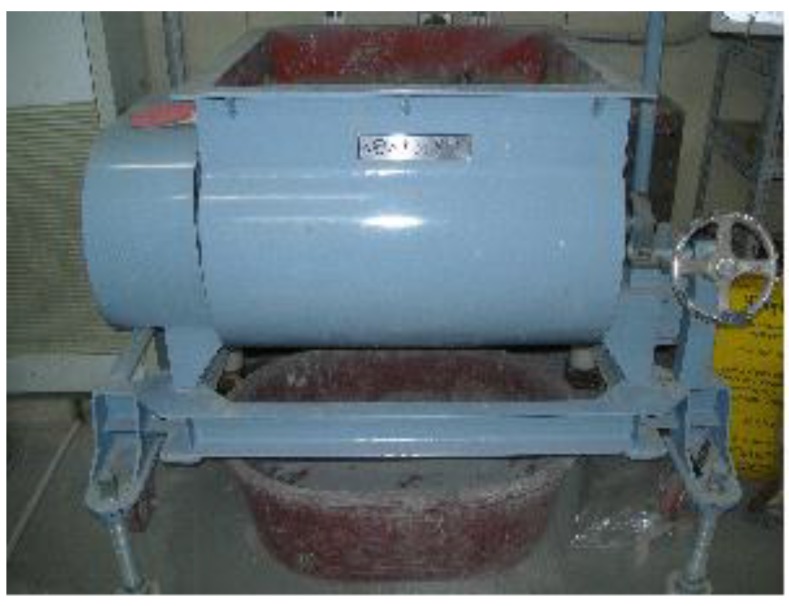
Two-shaft twin mixer.

For the concrete mixing, the two-shaft twin mixer shown in Figure 1 was used. Binder and aggregate were put into the mixer, and dry mixing was performed for 30 s. Water and chemical admixture were then added, and mixing was performed for 90 s.

**Table 1:** Experimental plan and mix proportion.

Binder Type	Adiabatic Specimen Volume (L)	W/B (%)	Slump (mm)	Air (%)	S/a (%)	Unit Weight (kg/m^3^)				Test Items
						**Water**	**Binder**	**Sand**	**Gravel**	
OPC	(1) 6 (2) 30 (3) 50	53.3	150 ± 25	4.5 ± 1.5	47	160	300	870	1011	—Adiabatic temperature rise (*Q*_∞_, *r*); —Correlation between the 6 L, 30 L and the 50 L adiabatic temperature rise tests
		40.0			46	160	400	814	985	
		32.0			45	160	500	759	956	
LPC		53.3			47	160	300	873	1014	
		40.0			46	160	400	817	989	
		32.0			45	160	500	763	961	
TBC		53.3			47	160	300	858	997	
		40.0			46	160	400	798	966	
		32.0			45	160	500	740	932	
EBC		53.3			47	160	300	858	997	
		40.0			46	160	400	798	966	
		32.0			45	160	500	740	932	

**Table 2:** Properties of the cement.

Material	Specific gravity	Blaine (cm^3^/g)	Ignition loss
Ordinary Portland cement	3.15	3475	2.15
Low heat Portland cement	3.22	3500	1.90
Ternary blended cement	2.88	3810	1.20
Early strength low heat blended cement	2.89	3802	0.07

**Table 3:** Properties of the aggregate.

Material	Type	Diameter (mm)	F.M	S.G
Fine aggregate	River sand	5	3.06	2.59
Coarse aggregate	Crushed stone	25	7.06	2.67

**Table 4:** Properties of the materials.

Material	Type	pH	S.G
Admixture 1	Polynaphthalene sulfonates based	6.0	1.20
Admixture 2	Polycarboxylic acid based	6.5	1.05

### 2.3. Adiabatic Temperature Rise Test

The temperature distribution of a concrete cross-section varies depending on the boundary conditions as well as the hydration heat of the cement. This can be numerically predicted by using the partial differential equation in Equation (1), which is derived from Fourier’s heat equation [19]:

(1) $$k\;\nabla^{2}T+q=\rho C\frac{\partial T}{\partial t}$$

where *k* = thermal conductivity (W/m·K); *C* = specific heat (kJ/kg·K); ρ = density (kg/m^3^); *T* = *T*(*x*, *y*, *z*, *t*) temperature of concrete (°C); *q* = *q*(*x*, *y*, *z*, *T*) rate of heat generated inside the body (kJ/m^3^·h).

In order to determine the internal heat generation of concrete (*q*), there is a method in which the hydration heat of cement is measured with a calorie meter and then converted into the heat generation of concrete. However, as the heat generation of concrete is influenced by many other factors, including the thermal properties of aggregate and cement, this method tends to underestimate the maximum adiabatic temperature rise and the rate of heat release. On the other hand, there is a method in which the heat generation of concrete is measured directly by performing an adiabatic temperature rise test. In this method, the concrete specimen is stored and monitored in the chamber where adiabatic condition is maintained automatically by controlling the boundary condition. This method has the advantages of making measurement simple, and the measured value can be applied to the temperature analysis of mass concrete as it stands [20].

**Figure 2:**
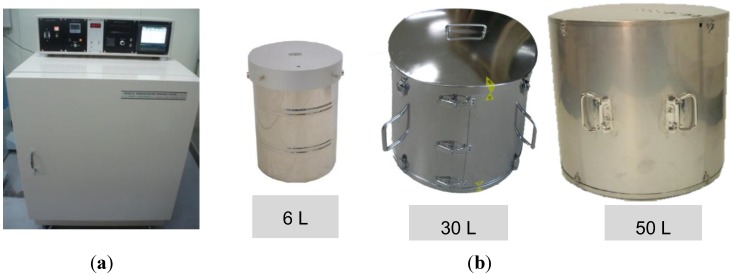
Adiabatic temperature rise apparatus. (**a**) Main body; (**b**) specimen vessel.

Figure 2 shows the apparatus (Tokyo-riko Company, Tokyo, Japan) for the adiabatic temperature rise test of concrete. Figure 2a shows the main body of the apparatus, which provides heat so that concrete can maintain adiabatic condition, and Figure 2b shows the specimen vessels, which are used to change the volume of adiabatic specimens. In order to measure the temperature of the adiabatic concrete specimens, a type of K thermocouple with a resolution limit capacity of 0.1 °C was used. Although platinum resistance temperature detectors (RTD) are able to measure a wide range of temperatures and are more accurate than the thermocouple, however, it is not adopted in commercial apparatus due to its high cost. The adiabatic performance of this apparatus provided by the manufacturer is within 0.05 K/day, which satisfies the recommendation of Rilem RC 119 sufficiently. 

The two parameter model shown in Equation (2) is adapted to represent the adiabatic temperature rise [21]. Using least square methods, the parameters were estimated. *Q*_∞_ represents the maximum temperature rise, and *r* represents the reaction factor:

(2) $$Q(t)=Q_{\infty }(1-e^{-rt})$$

where *Q* = temperature rise at time *t* (°C); *t* = time (day).

## 3. Results and Discussion

### 3.1. Influence of Binder Types on Adiabatic Temperature History

**Figure 3:**
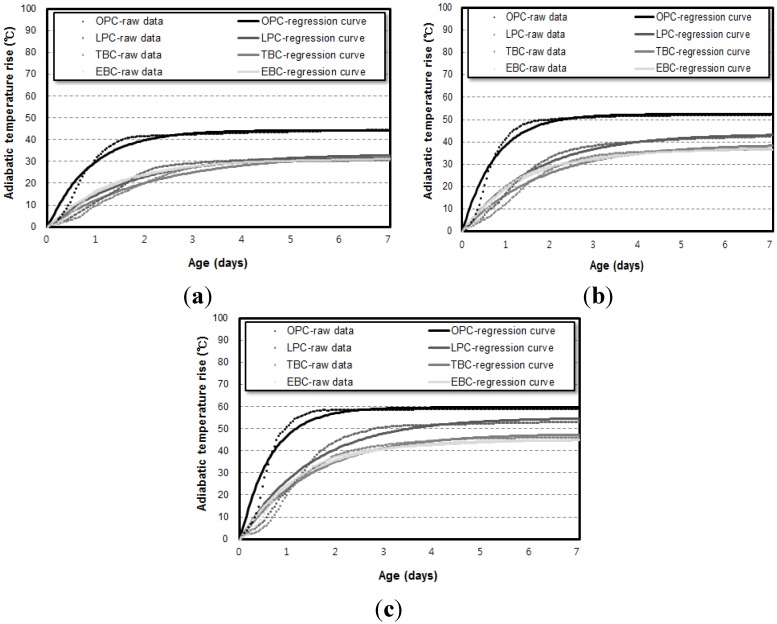
Results of the 50 L hadiabatic temperature rise test depending on binder types. (**a**) Unit weight of binder: 300 kg/m^3^; (**b**) unit weight of binder: 400 kg/m^3^; (**c**) unit weight of binder: 500 kg/m^3^.

**Figure 4:**
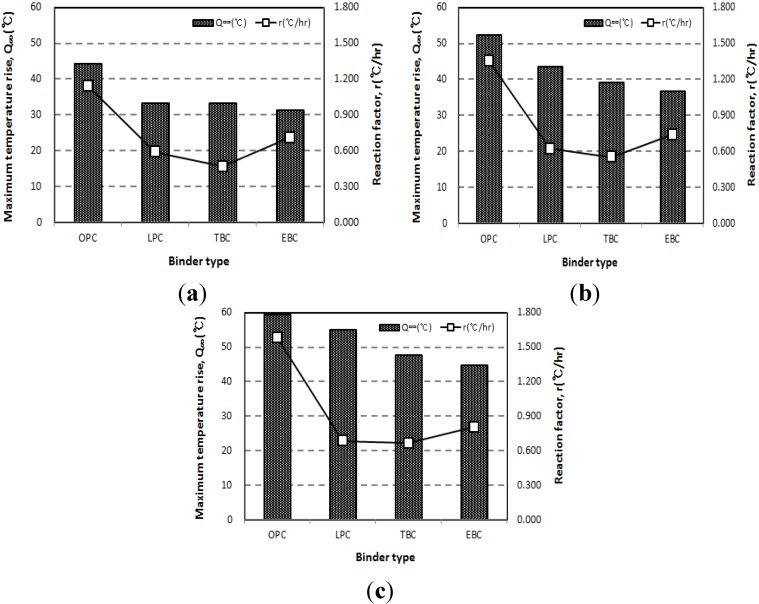
Results of the adiabatic temperature rise test for (**a**) unit weight of binder of 300 kg/m^3^; (**b**) unit weight of binder of 400 kg/m^3^; and (**c**) unit weight of binder of 500 kg/m^3^.

Figure 3 shows the adiabatic temperature rise curve of the 50 L specimens depending on binder types. Figure 4 shows the results with respect to the unit weight of binders. For every unit weight of binder, the maximum temperature rise (*Q*_∞_) was in the order of the OPC, LPC, TBC, and EBC mixtures from highest to lowest. The reaction factor (*r*) was in the order of the OPC, EBC, LPC, and TBC mixtures from highest to lowest.

When we compare the TBC and EBC, the maximum temperature rise was insignificant (5.7%–5.9%). However, the reaction factor of the EBC mixture was from 20.7% to 51.8% higher than that of the TBC mixture. Therefore, for the mass concrete applications, the fact that the EBC had a higher reaction factor than the TBC needs to be sufficiently taken into account.

**Figure 5:**
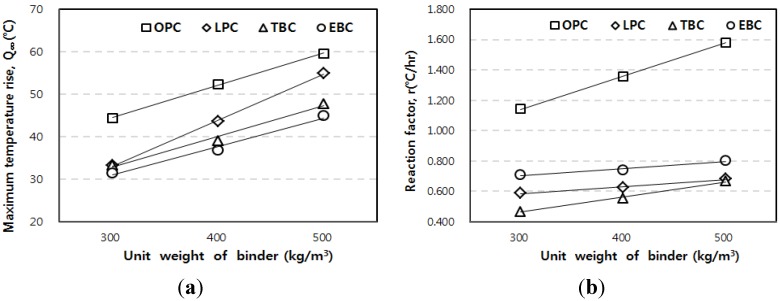
Results of the adiabatic temperature rise test for (**a**) maximum temperature rise; and (**b**) reaction factor.

Figure 5 shows the estimated parameters, which are shown with respect to the unit weight of binder. The maximum temperature rise (*Q*_∞_) increased as the unit weight of binder increased for every mixture. Especially for the LPC mixture, *Q*_∞_ increased at a relatively high rate with the increase in the unit weight of binder. As shown in Figure 5b, the reaction factor increased as the unit weight of binder increased for every mixture. However, the LPC, TBC, and EBC mixtures showed only small changes when the unit weight of binder increases.

### 3.2. Influence of Specimen Volume on Adiabatic Temperature History

**Figure 6:**
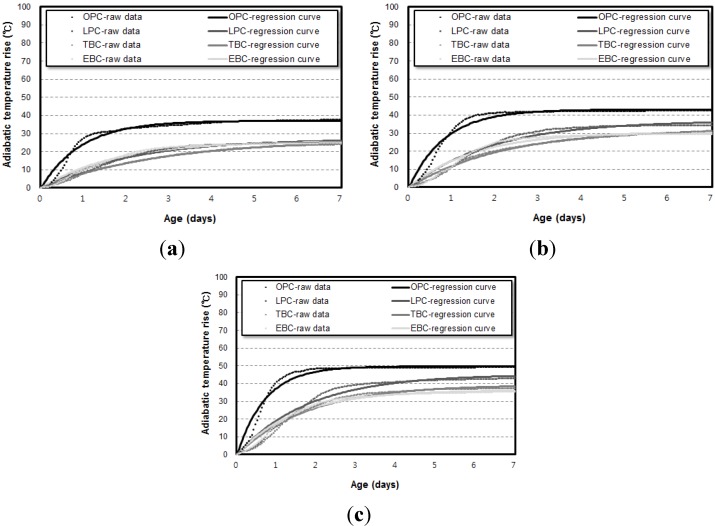
Amount results of the 6 L adiabatic temperature rise test depending on binder types. (**a**) Unit weight of binder: 300 kg/m^3^; (**b**) unit weight of binder: 400 kg/m^3^; (**c**) unit weight of binder: 500 kg/m^3^.

**Figure 7:**
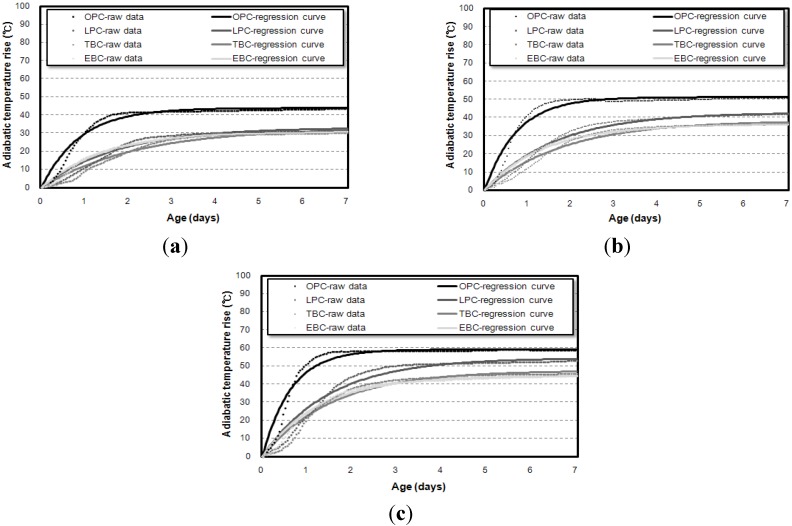
Results of the 30 L adiabatic temperature rise test depending on binder types. (**a**) Unit weight of binder: 300 kg/m^3^; (**b**) unit weight of binder: 400 kg/m^3^; (**c**) unit weight of binder: 500 kg/m^3^.

Figure 6 and Figure 7 show the results of the adiabatic temperature rise test for 6 L and 30 L specimens, respectively. At a glance, it can be observed that the maximum temperature rise increases when a higher volume of specimens is used.

**Figure 8:**
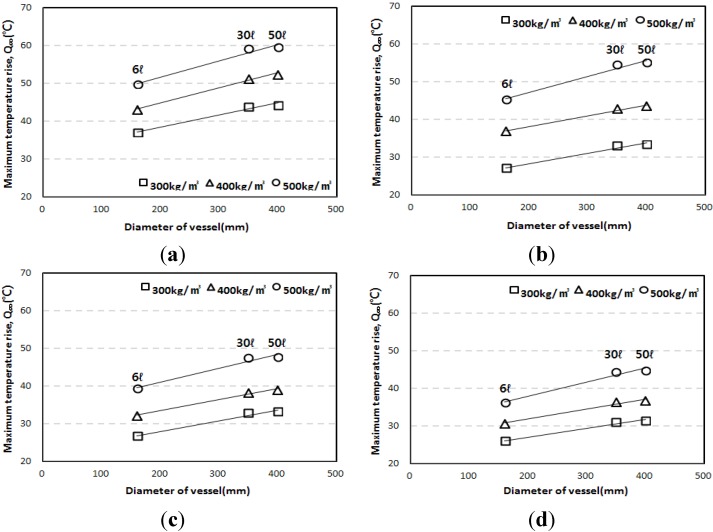
Maximum temperature rise obtained from the 6 L, 30 L, and 50 L specimen tests. (**a**) OPC; (**b**) LPC; (**c**) TBC; (**d**) EBC.

**Figure 9:**
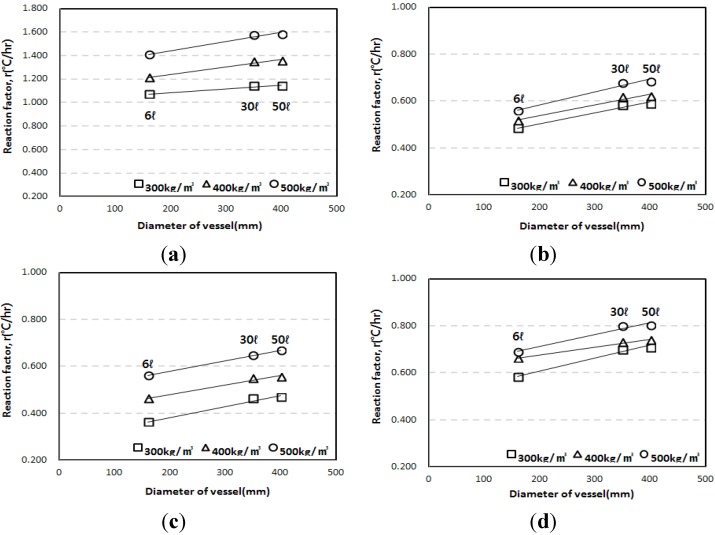
Reaction factor obtained from the 6 L, 30 L, and 50 L specimen tests. (**a**) OPC; (**b**) LPC; (**c**) TBC; (**d**) EBC.

Figure 8, which shows the maximum temperature rise depending on the diameter of the adiabatic specimen, proves this trend. Also, the maximum temperature rises of the 6 L specimens were smaller than those of others by 13.7%–19.8%. However, the results of 30 L and 50 L specimens showed only small differences. Figure 9 shows similar trends for the reaction factors, as the results for the 6 L specimen were smaller than those for the 30 L or 50 L specimens.

Ideally, the volume of specimens is not supposed to influence the adiabatic temperature history. However, test results shown in Figure 8 and Figure 9 indicate that the results vary with respect to the volume of specimens. The fact that the *Q*_∞_ and the *r* increased as the diameter of the adiabatic specimen increased is thought to be due to the characteristics of the adiabatic temperature rise test apparatus.

**Figure 10:**
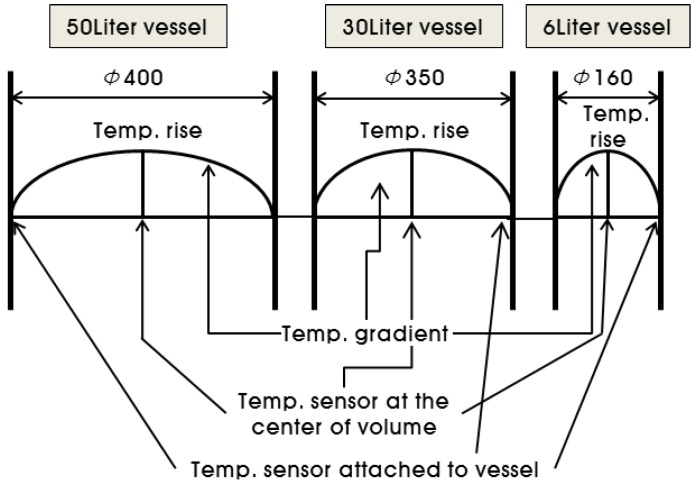
Comparison of the temperature gradients that are assumed to be linear.

As shown in Figure 10, the adiabatic temperature rise test apparatus simulates adiabatic conditions by measuring the temperature rise of the center part of the concrete using a sensor and by heating the outer vessel accordingly. Assuming that the binder and the aggregate are evenly mixed within the concrete, if the adiabatic control is perfect, the temperature of the concrete will be uniform across the entire volume. However, because of errors in temperature measurement and control, it is impossible to control the temperature of the outer vessel in real time so that it is identical to the temperature of the center part [17]. Thus, the apparatus simulates adiabatic conditions by controlling the temperature of the outer vessel. Thus, if the same apparatus is used for testing, the temperature gradient between the center part of concrete and the outer vessel would increase as the volume of the specimen decreases. This means that a test result with a larger specimen tends to have a smaller temperature loss. The 50 L vessel has a size of φ400 × h400 mm, the 30 L vessel has a size of φ350 × h350 mm, and the 6 L vessel has a size of φ160 × h300 mm. 

The test results also indicate that, as shown in Figure 8 and Figure 9, the *Q*_∞_ and the *r* increased in proportion to the diameter of the vessel; for the 6 L vessel with a diameter of 160 mm, the *Q*_∞_ and the *r* were the lowest because the largest heat loss occurred. For the 30 L and 50 L vessels with similar diameters (350 mm and 400 mm, respectively), the 50 L vessel had a slightly higher *Q*_∞_ and *r* than the 30 L vessel. Therefore, it is thought that the diameter of the measurement vessel influences the adiabatic temperature rise even though the test apparatus satisfies the requirement for adiabatic condition recommended by Rilem TC 119-TCE.

### 3.3. Correlation of the Adiabatic Temperature Rises and the Specimen Volume

**Figure 11:**
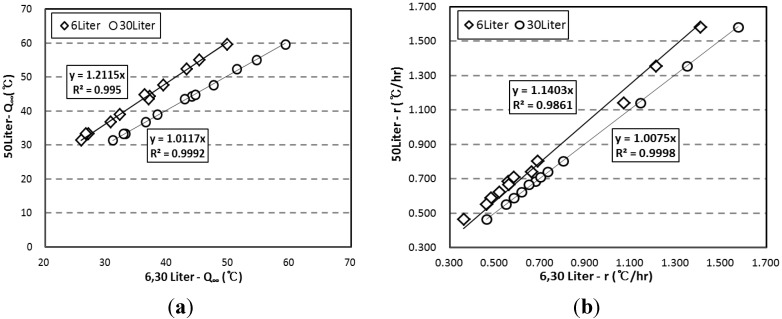
Correlation between the 6 L, 30 L, and 50 L adiabatic temperature rise test results for (**a**) maximum temperature rise; and (**b**) reaction factor.

Figure 11 shows the correlations for the results of the *Q*_∞_ and the *r* depending on adiabatic specimen volume. In Figure 11a, the regression lines were drawn using a *y*-intercept of 0. For the 6 L specimen, the regression function of *y* = 1.2115*x* was obtained, and the *R*-squared was 0.995, which indicated a very high correlation. For the 30 L specimen, the regression function of *y* = 1.0117*x* was obtained, and the *R*-squared was 0.999. Therefore, it was found that the *Q*_∞_ of the 50 L specimen was about 17.5% and 2.1% higher than those of the 6 L and 30 L specimens, respectively. Also, in Figure 11b, the regression lines were drawn using a *y*-intercept of 0, depending on vessel type. For the 6 L specimen, the regression function was *y* = 1.1403*x*, and the *R*-squared was 0.9861; and for the 30 L specimen, the regression function was *y* = 1.0075*x*, and the *R*-squared was 0.9998, which indicated a very high correlation, similar to the case of *Q*_∞_. Also, it was found that the *r* of the 50 L specimen was about 12.5% and 0.8% higher than those of the 6 L and 30 L specimens, respectively.

By using the proposed correlation, the adiabatic temperature characteristics of specimens with different volumes can be converted and interpreted with each other. For example, quality control of the specimens 50 L, 30 L, and 6 L can be performed using the proposed correlations. Also, when a small-scale test is performed in the laboratory, adiabatic temperature rise could be predicted with a high degree of accuracy based on the above correlations.

## 4. Conclusions

The results for the investigation into adiabatic temperature rise’s dependence on binder type and adiabatic specimen volume are as follows:

(1) The adiabatic temperature rise test showed that for every mixture, the *Q*_∞_ and the *r* increased in proportion to the unit weight of binder. Of the mixtures, the EBC mixture had the lowest *Q*_∞_ and the TBC mixture had the lowest *r*.
(2) Even though the experiment was conducted using an adiabatic calorimeter satisfying the minimum requirement of temperature loss, test results show that the volume of samples influences the adiabatic test results. For every mixture, the maximum temperature increase (*Q*_∞_) and reaction factor (*r*) of the 50 L specimens were about 17.5% and 12.5% higher than those of the 6 L specimens. However, there are only little differences (*Q*_∞_ 1.27%, *r* 1.30%) between the results of the 30 L and 50 L specimens. This proves that even a small temperature loss can affect the adiabatic temperature history of a small size of specimen. In this experiment, a 4 L sample size was not appropriate for an adiabatic temperature rise test.
(3) Based on the test results, correlations are proposed for the compensation of temperature loss with small-size specimens. By using the proposed correlation depending on adiabatic specimen volume, the adiabatic temperature rise of the 50 L specimen could be predicted based on the results of the 6 L and 30 L specimens. Therefore, it is thought that this correlation can be used as baseline data for on-site concrete quality control and research purposes.
